# Supplementary material for: 3D convolutional deep learning for nonlinear estimation of body composition from whole body morphology
Source: NPJ Digit Med. 2025 Feb 2;8:79. doi: 10.1038/s41746-025-01469-6 (PMC11788428; doi:10.1038/s41746-025-01469-6)
Supplement: Supplementary file 1 — Supplementary Material [file 41746_2025_1469_MOESM1_ESM.pdf]

SUPPLEMENTARY MATERIAL

Gaussian process regressions are well-known general algorithms not specific to this work. We summarize its mathematical context here for the reader's convenience.

A Gaussian process $f(x)$ is characterized by a multivariate Gaussian probability distribution $f(x) \sim N(\mu, \sigma^2\mathbf{K})$ for a feature vector **x**, where μ is the mean, σ^2 is the variance, and **K** is the symmetric covariance matrix where each entry $\mathbf{K}_{i,j} = K_{\mathbf{\Theta}}(x_i, x_j)$ for some kernel function $K_{\mathbf{\Theta}}$ parameterized by $\mathbf{\Theta}$ computed at all paired combinations of training data $\mathbf{x}_i, \mathbf{x}_j$. The kernel function $K_{\mathbf{\Theta}}$ is a measure of distance between two sample points x_i, x_j in kernel space and implies that inputs close together in kernel space should have similar predicted targets y_i, y_j. For our experiments, $K_{\mathbf{\Theta}}(\mathbf{x}_i, \mathbf{x}_j) = \theta_1(\mathbf{x}_i \cdot \mathbf{x}_j\ +\ \theta_2)^2\ +\ \theta_3$ for kernel parameters $\mathbf{\Theta} = \{\theta_1, \theta_2, \theta_3\}$.The parameters of the Gaussian process $\mu, \sigma^2, \mathbf{\Theta}$ are optimized via maximum likelihood estimation (MLE) of the probability density function (PDF) of the multivariate Gaussian for *n* observations:

$$L(\mathbf{y}|\mu, \sigma^2, \mathbf{\Theta}) = \frac{1}{\sqrt{(2\pi\sigma^2)^n|\mathbf{K}|}} \exp(-\frac{1}{2\sigma^2}(\boldsymbol{y} - \mathbf{1}\mu)^{\mathrm{T}}\mathbf{K}^{-1}(\boldsymbol{y} - \mathbf{1}\mu)) \qquad \text{(S1)}$$

where **1** is an *n* length vector of 1's. The parameters $\mu, \sigma^2, \mathbf{\Theta}$ are optimized by setting the derivative of the log-likelihood ln (L) to 0 and solving with standard methods:

$$\ln(L) = \frac{-1}{2\sigma^2}(\boldsymbol{y} - \mathbf{1}\mu)^{\mathrm{T}}\mathbf{K}^{-1}(\boldsymbol{y} - \mathbf{1}\mu)$$

$$\hat{\mu}, \hat{\sigma}^2, \hat{\mathbf{\Theta}}\ =\ \arg\max \ln(L) \qquad \text{(S2)}$$

Sampling from the optimized posterior distribution at $\mathbf{x}^*$ to calculate a prediction for regression is equivalent to determining the conditional distribution $P(f^*|\boldsymbol{y})$ where f^* is the Gaussian distribution describing the PDF of the target variable at input feature vector $\mathbf{x}^*$. The

predicted value is conventionally interpreted as the mean of the Gaussian f^*, which can be analytically derived as:

$$\mu^* = \hat{\mu} + \mathbf{k}^{*T}\mathbf{K}_{\hat{\boldsymbol{\theta}}}^{-1}(\boldsymbol{y} - \mathbf{1}\hat{\mu}) \quad \text{(S3)}$$

Where $\mathbf{k}^*$ is a vector containing all pairs $K_{\boldsymbol{\theta}}(\mathbf{x}^*, \mathbf{x}_i)$ for all *n* training observations $\mathbf{x}_i$, and $\hat{\mu}$, $\mathbf{K}_{\hat{\boldsymbol{\theta}}}^{-1}$ are the same optimized parameters as Eq. S2.

Supplementary Table 1 and 2 contains the population statistics of the Shape Up! Adults data for males and females respectively from which our body composition regression mappings were trained using ordinary least squares and Gaussian process regression.

Supplementary Table 1. Male participant statistics. Plus-minus values are standard deviation. p-value of 0.004 was considered significant after Bonferroni correction. All metrics were not significantly different between test and train. Body composition measurements were taken from DXA.

	**Male**					
	Train (N = 391)			Test (N = 181)		
	Mean ± SD	Min	Max	Mean ± SD	Min	Max
Age (Years)	44.92 ± 16.06	18	79	44.07 ± 16.15	18	79
Height (m)	1.76 ± 0.08	1.51	2.02	1.75 ± 0.07	1.55	1.91
Mass (kg)	88.42 ± 21.34	40.74	172.4	84.25 ± 18.91	40.77	135.58
BMI	28.44 ± 6.21	16.52	52.55	27.44 ± 5.55	16.96	45.82
Percent Fat	22.66 ± 6.86	9.03	47.69	22.07 ± 6.71	9.03	38.58
Lean Mass (kg)	67.48 ± 12.99	33.95	107.82	64.87 ± 11.64	33.95	93.1
Fat Mass (kg)	20.94 ± 10.67	5.01	66.48	19.39 ± 9.47	5.13	45.94
Visceral Fat (kg)	0.49 ± 0.27	0.16	1.64	0.5 ± 0.32	0.16	1.64
Leg Lean (kg)	10.97 ± 2.28	5.43	18.95	10.48 ± 1.99	5.43	14.74
Arm Lean (kg)	4.46 ± 1.05	2.05	8.33	4.26 ± 0.98	2.05	7.38
Trunk Lean (kg)	32.36 ± 6.4	15.95	51.16	31.24 ± 5,82	15.95	48.02
Trunk Fat (kg)	10.5 ± 6.15	1.76	34.12	9.72 ± 5.71	1.97	26.25
Leg Fat (kg)	3.41 ± 1.71	0.89	11.86	3.12 ± 1.44	0.85	9.02
Arm Fat (kg)	1.25 ± 0.68	0.29	4.34	1.15 ± 0.61	0.29	3.72

FMI	6.74 ± 3.37	1.68	20.78	6.32 ± 3.01	1.91	15.49
FFMI	21.72 ± 3.53	14.18	35.65	21.16 ± 3.23	14.18	30.72

Supplementary Table 2. Female participant statistics. Plus-minus values are standard deviation. p-value of 0.004 was considered significant after Bonferroni correction. All metrics were not significantly different between test and train. except for female height, denoted by the asterisk, which is not a predicted measurement. Body composition measurements were taken from DXA.

	**Female**					
	Train (N = 457)			Test (N = 239)		
	Mean ± SD	Min	Max	Mean ± SD	Min	Max
Age (Years)	46.24 ± 16.13	18	89	47.53 ± 16.71	18	89
Height (m)	1.62 ± 0.07	1.44	1.80	1.61 ± 0.07 *	1.44	1.76
Mass (kg)	72.43 ± 20.93	35.44	153.05	69.39 ± 19.60	35.44	153.05
BMI	27.48 ± 7.65	14.16	51.86	26.81 ± 7.05	14.16	51.86
Percent Fat	34.06 ± 7.88	12.63	53.3	33.78 ± 7.44	17.18	53.3
Lean Mass (kg)	46.49 ± 9.48	28.56	80.37	44.91 ± 9.42	28.56	80.37
Fat Mass (kg)	25.85 ± 12.72	6.3	72.68	24.39 ± 11.38	6.88	72.68
Visceral Fat (kg)	0.45 ± 0.31	0.06	1.37	0.43 ± 0.3	0.05	1.22
Leg Lean (kg)	7.48 ± 1.73	4.25	14.03	7.21 ± 1.78	4.42	13.19
Arm Lean (kg)	2.42 ± 0.57	1.31	4.42	2.34 ± 0.58	1.44	4.63
Trunk Lean (kg)	23.13 ± 4.91	13.71	41.59	22.26 ± 4.72	13.71	41.14
Trunk Fat (kg)	11.94 ± 6.75	2.37	35.6	11.27 ± 6.21	2.48	35.6
Leg Fat (kg)	4.81 ± 2.23	1.23	12.92	4.54 ± 1.98	1.23	12.34
Arm Fat (kg)	1.67 ± 0.99	0.28	6.08	1.55 ± 0.84	0.4	6.08
FMI	9.82 ± 4.79	2.01	26.57	9.44 ± 4.24	2.75	24.68
FFMI	17.6 ± 3.28	11.43	29	17.33 ± 3.16	10.93	28.88

Supplementary Table 3 shows differences between a linear shape model (PCA) and a nonlinear shape model (3DAE) when the regression method and dimensionality are held constant. 3DAE-GPR for males trained on the bottleneck layer with 4284 features achieved lower RMSEs than PCA-GPR with the exact same parameter count, while the same was not true

for females. Coefficients of determination (R^2) for the best 3DAE-GPR models were greater than or equal to 0.86 for all predicted variables.

Supplementary Table 3. Body composition prediction RMSEs comparison between 3DAE-GPR and PCA-GPR holding all variables constant except for shape feature type. 3DAE was trained with a d=4284 sized bottleneck. The bottleneck layer (with a size 4284 feature vector) was used to train the GPR to body composition. PCA-GPR was trained with 4284 PCA components, the same feature vector size as 3DAE-GPR. Males exhibited lower RMSE when 3DAE was used as the shape feature extractor, while females exhibited comparable performance with mixed results. 3DAE features are informative for males but not necessarily so for females.

	**RMSEs**			
	**Male PCA-GPR (d=4284)**	**Male 3DAE-GPR (7x612)**	**Female PCA-GPR (d=4284)**	**Female 3DAE-GPR (7x612)**
Fat Mass (kg)	2.22	2.15	1.9	2.03
Lean Mass (kg)	2.22	2.15	1.9	2.03
Visc. Fat (kg)	0.11	0.11	0.11	0.11
Arm Lean (kg)	0.3	0.28	0.2	0.22
Leg Lean (kg)	0.52	0.5	0.46	0.45
Percent Fat (%)	2.68	2.56	2.85	2.89
Trunk Fat (kg)	1.18	1.15	1.09	1.13
Trunk Lean (kg)	1.22	1.18	1.08	1.1
Arm Fat (kg)	0.22	0.2	0.2	0.21
Leg Fat (kg)	0.48	0.48	0.47	0.49
